# Structure of a mosaic hybrid zone between the field crickets *Gryllus firmus* and *G. pennsylvanicus*

**DOI:** 10.1002/ece3.514

**Published:** 2013-03-07

**Authors:** Erica L Larson, C Guilherme Becker, Eliana R Bondra, Richard G Harrison

**Affiliations:** Department of Ecology and Evolutionary Biology, Cornell UniversityIthaca, NY, USA

**Keywords:** Habitat association, introgression, reproductive isolation, speciation

## Abstract

Hybrid zones provide insight into the nature of species boundaries and the evolution of barriers to gene exchange. Characterizing multiple regions within hybrid zones is essential for understanding both their history and current dynamics. Here, we describe a previously uncharacterized region of a well-studied hybrid zone between two species of field crickets, *Gryllus pennsylvanicus* and *G. firmus*. We use a combination of mitochondrial DNA sequencing, morphological data, and modeling of environmental variables to identify the ecological factors structuring the hybrid zone and define patterns of hybridization and introgression. We find an association between species distribution and natural habitat; *Gryllus pennsylvanicus* occupies natural habitat along forest edges and natural clearings, whereas *G. firmus* occupies more disturbed areas in agricultural and suburban environments. Hybridization and introgression occur across patch boundaries; there is evidence of substantial admixture both in morphological characters and mtDNA, over a broad geographic area. Nonetheless, the distribution of morphological types is bimodal. Given that F_1_ hybrids are viable and fertile in the lab, this suggests that strong pre-zygotic barriers are operating in this portion of the hybrid zone.

## Introduction

Hybrid zones have been described as ‘natural laboratories for evolutionary studies’ (Hewitt 1988; Barton and Hewitt 1989) and ‘windows on evolutionary process’ (Harrison 1990, 1993). They are places where diverged lineages meet and interact, providing insight into the genetic architecture of speciation and the evolutionary forces that shape divergence (Barton and Hewitt 1985; Harrison 1990; Payseur 2010). Hybrid zone studies have demonstrated that species boundaries are semipermeable and that permeability varies across the genome (Key 1968; Barton and Hewitt 1981; Harrison 1986, 1990; Wu 2001; Nosil et al. 2009; Payseur 2010). Genomic regions that contain alleles contributing to reproductive isolation will either prevent the formation of hybrids or decrease hybrid viability or fertility, restricting introgression. Recombination over multiple generations breaks up parental genomes and some segments of the genome can then be freely exchanged between species. From studies of differential introgression, we can identify genomic regions that are under selection, estimate the strength of that selection, and ultimately link some of those regions to reproductive barriers (Harrison 1990; Payseur 2010).

Many hybrid zones may be tension zones, in which parental types persist because of selection against hybrid genotypes, independent of environment (Barton and Hewitt 1985). However, hybrid zones may also be maintained by environmental selection favoring different parental forms in different ecological settings. In the latter case, hybrid zones can be clinal, with parental forms favored on either side of an ecotone and hybridization occurring in the center (Endler 1977). Alternatively, hybrid zones can be mosaic, with parental forms patchily distributed across heterogeneous habitat and hybridization occurring across patch boundaries or in intermediate habitats (Harrison 1986; Harrison and Rand 1989; Rand and Harrison 1989; Ross and Harrison 2002; but see Searle 1993). In a heterogeneous landscape, hybrid zones may exhibit a mix of different dynamics (e.g., *Bombina* hybrid zone; Szymura and Barton 1991; Vines et al. 2003; Yanchukov et al. 2006), and as a consequence, reproductive barriers and patterns of introgression may vary geographically (Teeter et al. 2010). Therefore, characterizing multiple transects or regions is essential for understanding both hybrid zone history and hybrid zone dynamics.

Here, we describe a previously uncharacterized region of a well-studied hybrid zone between two species of North American field crickets, *Gryllus pennsylvanicus* and *G. firmus*. The hybrid zone has been carefully characterized in Virginia and Connecticut, and reproductive barriers are known to vary between these two regions. This study examines patterns of variation in Pennsylvania, compares these patterns with those seen elsewhere, and investigates what ecological factors maintain the structure of the hybrid zone.

### Field cricket hybrid zone

**Figure 1:**
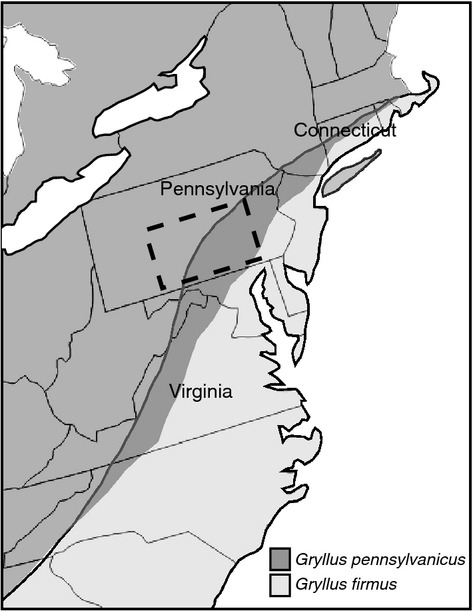
Geographic ranges of *Gryllus pennsylvanicus* (medium gray) and *G. firmus* (light gray) in the eastern United States. The hybrid zone is represented by the area of overlap in the two ranges, indicated by the dark gray shading. Two previously described regions of the hybrid zone in Connecticut and Virginia are labeled and the rectangle indicates the study area in Pennsylvania.

The known hybrid zone between *G. pennsylvanicus* and *G. firmus* stretches from southern Connecticut to Virginia along the eastern slopes of the Appalachian, Blue Ridge, and Northern Highland Mountains (Harrison and Arnold 1982) (Fig. 1). The glacial history of the northeastern United States and the distribution of *Gryllus* mitochondrial DNA (mtDNA) haplotypes provide strong evidence that the hybrid zone formed as a result of secondary contact between lineages that diverged in allopatry (Harrison et al. 1987; Willett et al. 1997; Maroja et al. 2009a). *Gryllus pennsylvanicus* extends west from the Appalachian and Blue Ridge Mountains and through the mountains to the south. *Gryllus firmus*, also known as the beach cricket, occurs to the east of the Appalachian Mountains throughout the Piedmont, coastal plain, and along beaches south into Florida (Alexander 1957, 1968; Harrison and Arnold 1982). Both species occupy grassy, disturbed habitats and can be found under rocks, debris or clumps of vegetation. Both species are univoltine in the north; females lay eggs in the soil, eggs diapause over the winter, hatch in the spring, and adults emerge in late summer or early fall (Fulton 1952). In the south, *G. firmus* is multivoltine and females lay both diapause and non-diapause eggs (Fulton 1952; Alexander 1968; Walker 1980).

**Figure 2:**
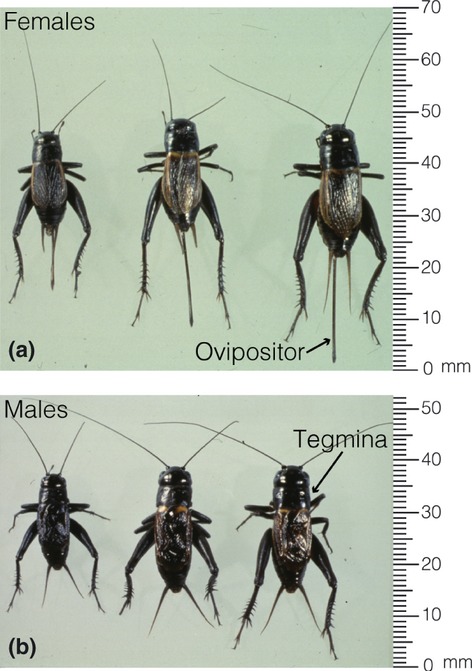
Morphological variation in *Gryllus pennsylvanicus* and *G. firmus*. Representative crickets of *G. pennsylvanicus* (left), *G. firmus* (right), and intermediate individuals (middle): females (a) and males (b). Arrows indicate the female ovipositor and male tegmen. *Gryllus firmus* are typically larger, with relatively longer ovipositors and lighter tegmina.

The two cricket species diverged about 200,000 ya (Willett et al. 1997; Maroja et al. 2009a). They are very similar morphologically and were considered part of a single variable species until the 1950s (Fulton 1952; Alexander 1957). *Gryllus pennsylvanicus* is, on average, a smaller cricket, with darker tegmina (modified leathery front wing) and a relatively shorter ovipositor (Fig. 2). Calling songs of the two species (used to attract females at long distances) are very similar, but have slightly different pulse and chirp rates; courtship songs (used to initiate mating) are identical (Alexander 1957; Doherty and Storz 1992). Despite these similarities, there is evidence of behavioral isolation, with females of both species reluctant to mate with heterospecific males in the laboratory (Maroja et al. 2009b). The crickets are also isolated by post-mating pre-zygotic barriers in one direction; *G. firmus* females mated with *G. pennsylvanicus* males lay few eggs, none of which are fertilized (Harrison 1983; Maroja et al. 2009b; Larson et al. 2012).

In Virginia, the two species are temporally isolated because of differences in development time, with *G. firmus* adults emerging later in the fall (Harrison and Arnold 1982; Harrison 1985). In Connecticut, adults emerge synchronously, but are associated with different soil types; *Gryllus firmus* on sandy soils and *G. pennsylvanicus* on loamy soils (Harrison 1986; Rand and Harrison 1989; Ross and Harrison 2002). What maintains this soil association remains unclear; females of both species prefer to oviposit in loamy soils in the laboratory (Ross 2000) and the viability of overwintering diapause eggs appears to be independent of soil type (Ross and Harrison 2006).

Although multiple barriers isolate these species and very few F_1_ hybrids are present within the hybrid zone, evidence for introgression is clear (Harrison and Bogdanowicz 1997; Ross and Harrison 2002). Some barriers appear to be consistent across different regions of the hybrid zone (assortative mating, one-way fertilization incompatibility), whereas other barriers vary from one ecological setting to another (development time, soil association). Documenting patterns of species distributions and their ecological context build the foundation for comparing patterns of introgression among different regions of the hybrid zone. Here, we describe patterns of variation in a previously uncharacterized region of the hybrid zone in south-central Pennsylvania.

## Materials and Methods

### Cricket sampling

In the fall of 2010, we collected 104 crickets from nine localities in the northeastern range of *G. firmus* and G*. pennsylvanicus*. To these, we added 26 crickets from four localities collected by Maroja et al. (2009a). We also used mitochondrial DNA (mtDNA) sequence data for 98 crickets from 28 localities described in Willett et al. (1997) and Maroja et al. (2009a) (Table S1). These samples provide a broad geographic context for analyzing the distribution of cricket mtDNA haplotypes.

In late summer/fall of 2008 and 2010, we collected 877 crickets from 88 localities within a small region of the hybrid zone in south-central Pennsylvania (Table 1). Collection localities span the transition from the Appalachian Mountains into the Great Appalachian Valley. In this region, the Appalachians form a series of continuous ridges and intervening valleys that can range in elevation from 100 m to 650 m above sea level (elevation can change from 250 m to 570 m over only 1.8 km). The ridges are broken by several narrow and dramatic gaps where the Susquehanna River and other small waterways cross the mountains. The Great Appalachian valley is an extended chain of lowlands bounded by the Appalachian Mountains to the west and the Blue Ridge Mountains and Northern Highlands to the east, and includes the Shenandoah Valley in northern Virginia. To the east of the Blue Ridge Mountains are the lowlands of the Piedmont and the coastal beaches. There is a large gap in the eastern ridge of mountains between the Reading Prong (near collecting locality I) and South Mountain (collecting locality AJ) through which the Susquehanna River passes, connecting the Great Appalachian Valley with the Piedmont region.

**Table 1:** Collecting localities for crickets in the Pennsylvania portion of the hybrid zone.

Locality	ID	Species	*N*	*N*^†^	Lat (N)	Long (W)	Ele (m)	Habitat	Date collected
Echo Valley	A	*Gp*	4	–	40^o^35′33″	76^o^24′36″	205	Rocks/forest clearing	2008
Outwood	B	*Gp*	2	–	40^o^31′38″	76^o^28′24″	154	Rocks/wooded roadside	2008
Indiantown Gap	C	*Gp*	8	5	40^o^26′08″	76^o^35′54″	156	Rocks/forest clearing stream	2008
Quentin	D	*Gp*	19	9	40^o^15′51″	76^o^26′08″	223	Rocks/wooded roadside	2008
Cornwall	E	*Gp*	3	–	40^o^16′36″	76^o^24′39″	172	Rocks/edge of cornfield	2008
Schaefferstown	F	*Gp*	5	–	40^o^17′37′	76^o^17′52″	178	Rocks/grassy field	2008
Womelsdorf	G	Admix (*Gp*)	5	–	40^o^23′24″	76^o^11′54″	146	Trash pile/grassy roadside	2008
Rehrersburg	H	*Gp*	7	7	40^o^26′57″	76^o^13′58″	169	Burrows/grassy slope	2008
Reading	I	Admix	12	12	40^o^22′51″	76^o^01′46″	104	Rocks/forest clearing	2008
Nottingham	J	Admix	10	10	39^o^44′15″	76^o^02′48″	111	Rocks/forest clearing	2008
Nottingham	K	Admix (*Gp*)	3	3	39^o^44′32″	76^o^02′02″	134	Trash pile/city park	2008
Holtwood	L	Admix (*Gp*)	8	8	39^o^48′49″	76^o^19′42″	48	Rocks/forest clearing	2008
Marysville	M	*Gp*	4	–	40^o^20′28″	76^o^54′36″	92	Rocks/shoreline	2008
Millersburg	N	Admix (*Gp*)	5	5	40^o^32′07″	76^o^57′59″	112	Rocks/wooded beach	2008
Bloomsburg	O	Admix (*Gp*)	4	4	40^o^58′39″	76^o^28′10″	142	Rocks/wooded campground	2008
Cattawissa	P	Admix (*Gp*)	3	–	40^o^56′55″	76^o^30′53″	145	Rocks/wooded roadside	2008
Shamokin Dam	Q	*Gp*	3	–	40^o^51′22″	76^o^48′32″	133	Rocks/boat launch	2008
Northumberland	R	*Gp*	19	9	40^o^53′02″	76^o^48′16″	202	Trash cans/forest clearing	2008
Etters	S	Admix (*Gf*)	1	–	40^o^09′00″	76^o^44′58″	89	Rocks/grassy field in park	2008
York Haven	T	Admix (*Gf*)	1	–	40^o^06′42″	76^o^42′36″	76	Rocks/wooded shoreline	2008
Mt Wolf	U	Admix	21	13	40^o^03′48″	76^o^42′34″	123	Rocks/grassy field slope	2008
York	V	*Gf*	1	1	39^o^58′59″	76^o^44′00″	116	Trash cans/motel parking lot	2008
York	W	Admix (*Gf*)	1	1	39^o^58′01″	76^o^46′38″	126	Trash cans/gas station	2008
Mt Royal	X	Admix (*Gf*)	4	4	40^o^02′35″	76^o^53′45″	115	Rocks/grassy roadside slope	2008
Pinchot Lake	Y	*Gp* & *Gf*	5	5	40^o^04′06″	76^o^54′32″	146	Rocks/wooded boat launch	2008
Dillsburg	Z	Admix	6	5	40^o^05′18″	77^o^01′19″	219	Rocks/wooded roadside	2008
Locust Point	AA	*Gp*	3	–	40^o^11′02″	77^o^03′19″	151	Rocks/grassy slope	2008
Wertzville	AB	*Gp*	3	–	40^o^16′30″	77^o^02′46″	140	Rocks/wooded roadside	2008
Goodhope	AC	Admix (*Gp*)	5	–	40^o^17′17″	77^o^00′11″	135	Rocks/wooded roadside	2008
Hummelstown	AD	Admix (*Gp*)	3	3	40^o^15′43″	76^o^41′18″	128	Rocks/edge of cornfield	2008
Harper Tavern	AE	Admix (*Gp*)	5	5	40^o^24′17″	76^o^34′32″	118	Rocks/grassy roadside slope	2008
Jonestown	AF	Admix (*Gp*)	10	5	40^o^24′55″	76^o^29′51″	141	Rocks/grassy roadside slope	2008
Carlisle	AG	Admix (*Gp*)	6	1	40^o^12′21″	77^o^16′22″	156	Rocks/grassy roadside slope	2008
Newville	AH	*Gp*	1	–	40^o^12′17″	77^o^24′46″	179	Rocks/grassy roadside slope	2008
Newville	AI	Admix	5	5	40^o^08′21″	77^o^21′56″	187	Rocks/pasture	2008
Michaux Forest	AJ	*Gp*	7	5	40^o^03′26″	77^o^17′46″	364	Rocks/wooded roadside	2008
Michaux Forest	AK	*Gp*	7	6	39^o^58′08″	77^o^22′46″	500	Rocks/wooded roadside	2008
Gettysburg	AL	Admix (*Gf*)	6	4	39^o^52′40″	77^o^14′38″	198	Rocks/grassy roadside slope	2008
Emmitsburg*	AM	Admix	9	7	39^o^42′11″	77^o^19′00″	125	Trash/freeway onramp	2008
Carroll Valley	AN	*Gp*	5	4	39^o^44′18″	77^o^23′33″	180	Rocks/wooded roadside	2008
Rouzerville	AO	Admix	5	3	39^o^44′17″	77^o^31′11″	557	Trash/parking lot	2008
Waynesboro	AP	Admix (*Gf*)	2	1	39^o^45′47″	77^o^35′52″	195	Rocks/grassy roadside slope	2008
Mercersburg	AQ	Admix	7	5	39^o^49′18″	77^o^53′56″	190	Rocks/cemetery lawn	2008
Charlestown	AR	*Gp* & *Gf*	5	5	39^o^52′15″	77^o^57′12″	269	Rocks/forest clearing	2008
Fort Loudon	AS	Admix (*Gf*)	5	5	39^o^54′41″	77^o^54′19″	186	Rocks/wooded roadside	2008
Saint Thomas	AT	Admix (*Gf*)	1	1	39^o^54′24″	77^o^50′24″	212	Rocks/parking lot	2008
**Saint Thomas**	**AU**	Admix	20	5	39^o^53′47″	77^o^47′02″	164	Concrete blocks/grassy field	2010
**Saint Thomas**	**AV**	Admix	21	–	39°53′06″	77°49′07″	196	Concrete blocks/churchyard	2010
**Fort Loudon**	**AW**	Admix	16	15	39^o^54′49″	77^o^54′19″	192	Trash cans/city park	2010
Big Mountain	AX	Admix	25	14	39^o^55′44″	77^o^57′18″	698	Rocks/forest clearing	2010
**Fayetteville**	**AY**	Admix	24	–	39^o^55′09″	77^o^33′18″	237	Woodpile/grassy field	2010
**South Mountain**	**AZ**	Admix	27	15	39^o^50′37″	77^o^28′40″	510	Concrete blocks/churchyard	2010
Quincy	BA	Admix (*Gf*)	9	5	39^o^48′38″	77^o^34′08″	235	Concrete blocks/churchyard	2010
Five Forks	BB	Admix	14	5	39^o^47′56″	77^o^36′37″	228	Boards/churchyard	2010
**Milnor**	**BC**	Admix	27	–	39^o^45′48″	77^o^46′04″	179	Boards/city park	2010
**Bino**	**BD**	Admix (*Gf*)	10	–	39^o^45′57″	77^o^47′49″	171	Woodpile/churchyard	2010
Shimpsown	BE	*Gp* & *Gf*	2	–	39°47′17″	77°50′34″	147	Trash pile/grassy field	2010
Big Mountain	BF	*Gp*	3	–	39°59′25″	77°55′51″	382	Rocks/forest clearing	2010
**Scotland**	**BG**	Admix	30	5	39^o^56″51″	77^o^33′41″	258	Woodpile/churchyard	2010
**Fayetteville**	**BH**	Admix (*Gp*)	10	–	39^o^54′21″	77^o^31′51″	264	Rocks/wooded roadside	2010
Cashtown	BI	Admix (*Gp*)	9	–	39^o^53′07″	77^o^22′04″	267	Rocks/wooded roadside	2010
**Michaux Forest**	**BJ**	Admix (*Gp*)	5	–	39^o^51′36″	77^o^26′18″	482	Rocks/wooded roadside	2010
Charmian	BK	*Gp* & *Gf*	12	12	39^o^44′22″	77^o^28′11″	416	Boards/churchyard	2010
**Chambersburg**	**BL**	Admix	28	–	39^o^54′46″	77^o^42′16″	229	Trash cans/city park	2010
New Franklin	BM	Admix	11	-	39^o^52′48″	77^o^38′18″	219	Woodpile/churchyard	2010
New Franklin	BN	Admix	12	5	39^o^52′07″	77^o^38′09″	248	Boards/churchyard	2010
**Pond Bank**	**BO**	Admix	23	5	39^o^52′21″	77^o^32′36″	272	Rocks/churchyard	2010
Kauffman	BP	Admix	26	5	39^o^50′04″	77^o^42′01″	195	Boards/city park	2010
**Cashtown**	**BQ**	Admix	32	5	39^o^52′03″	77^o^43′41″	201	Concrete blocks/churchyard	2010
**Saint Thomas**	**BR**	Admix	8	–	39^o^53′59″	77^o^45′32″	197	Boards/churchyard	2010
Markes	BS	Admix (*Gf*)	4	–	39^o^52′29″	77^o^52′20″	172	Boards/city park	2010
**Sylvan**	**BT**	*Gp* & *Gf*	25	-	39^o^45′16″	78^o^01′27″	154	Boards/lumberyard	2010
Lemasters	BU	Admix	18	16	39^o^51′23″	77^o^51′41″	174	Trash cans/city park	2010
**Williamson**	BV	Admix	20	15	39^o^51′12″	77^o^48′04″	165	Trash cans/city park	2010
**Chambersburg**	**BW**	Admix (*Gf*)	10	–	39^o^57′41″	77^o^43′48″	170	Trash cans/city park	2010
**Edenville**	**BX**	Admix	22	5	39^o^57′35″	77^o^47′54″	219	Boards/churchyard	2010
Charlestown	BY	*Gp*	2	–	39^o^52′10″	77^o^57′23″	339	Rocks/forest clearing	2010
Harrisonville	BZ	Admix (*Gp*)	5	5	39^o^59′16″	78^o^03′45″	241	Concrete blocks/churchyard	2010
Bedford	CA	Admix (*Gp*)	4	4	40^o^05′03″	78^o^31′31″	361	Trash cans/city park	2010
Osterburg	CB	Admix (*Gp*)	14	5	40^o^10′25″	78^o^31′42″	354	Grass clumps/grassy field	2010
PA Turnpike	CC	*Gf*	3	–	40^o^01′05″	78^o^11′23″	538	Woodpile/grassy roadside	2010
Saluvia	CD	Admix	10	9	39^o^59′05″	78^o^06′36″	333	Grass clumps/grassy field	2010
Andover	CE	Admix	15	15	39^o^55′43″	78^o^06′28″	306	Trash cans/churchyard	2010
Needmore	CF	Admix (*Gp*)	7	7	39^o^49′40″	78^o^15′15″	273	Burrows/roadside slope	2010
**Breezewood**	**CG**	Admix (*Gf*)	17	5	39^o^59′58″	78^o^14′14″	391	Concrete blocks/hotel lawn	2010
**Everett**	**CH**	Admix	7	6	40^o^00′31″	78^o^22′35″	322	Burrows/bank along river	2010
Hopewell	CI	Admix	17	16	40^o^08′29″	78^o^19′59″	318	Burrows/roadside slope	2010
Roaring Spring	CJ	Admix (*Gp*)	10	5	40^o^20′27″	78^o^52′50″	417	Rocks/grassy roadside slope	2010
**Total**			**877**	**375**					

* Site located in Maryland.

*Gp* = *Gryllus pennsylvanicus*, *Gf* = *G. firmus*, Admix = hybrid or backcross crickets, *N* = total number of crickets collected at each locality, *N*^†^ = number of crickets genotyped for mitochondrial DNA, Lat = latitude, Long = longitude, Ele = elevation measured in meters above sea level. Habitat includes both the type of substrate crickets that were collected from under (e.g., rocks, trash, woodpiles, etc.) and the type of surrounding habitat (e.g., forest, field, etc.). Bold font indicates sites in which long-winged crickets were found.

In general, the mountains are heavily forested (poor cricket habitat), but have some natural clearings and are dissected by roadways and water gaps. The mountain valleys are typically moderately populated farmland, whereas the relevant portion of the Great Appalachian valley (Lehigh, Lebanon, and Cumberland valleys) is relatively heavily populated, and primarily agricultural or suburban.

Crickets were collected by hand, and maintained in plastic containers with food (cat food and rabbit food), water vials, and shelter (paper towels) prior to being frozen at −80°C. The majority of crickets were collected as adults, but in some cases, crickets were collected as late instar nymphs. Nymphs were allowed to mature in the laboratory before freezing.

### Mitochondrial DNA

We sequenced the mtDNA gene cytochrome oxidase I (COI) for a total of 130 crickets from 13 localities across the hybrid zone and 119 crickets from 31 localities within the Pennsylvania hybrid zone. We isolated genomic DNA from a single femur using the DNeasy tissue kit (Qiagen, Valencia, CA). Locus specific primers were used to amplify a 1.9-kb fragment of the mitochondrial DNA gene cytochrome oxidase I (COI), the adjacent tRNA, and a portion of cytochrome oxidase II (COII): *G. veletis* COI F (102) (5′- ACCCCCATCATTAACCCTTTTA -3′)(Maroja et al. 2009a) and Eva/3372 (1885) (5′- GAGACCATTACTTGCTTTCAGTCATCT -3′) (Simon et al. 1994). A set of internal primers were designed from *G. firmus* mtDNA sequence for samples with shorter sequence length: cricketCOI.595 (5′- ATTTACGGTTGGAATAGATGTTGATACCC -3′) and cricketCOI.1270 (5′- GAAGCTTAAATTCATCGCACTTTTCTG -3′). DNA was amplified using polymerase chain reaction (PCR) in 10 μL reactions containing: 1 μL genomic DNA, 2 mmol/L MgCl_2_, 0.2 mmol/L dNTPs, 0.2 μmol/L of each forward and reverse primer, and 0.1 μL (0.5 U) Platinum Taq polymerase (Invitrogen, San Diego, CA) in 1 × PCR buffer (20 mmol/L Tris-HCL, pH 8.4, 50 mmol/L KCl). PCR was performed using an initial denaturation of 95°C for 2 min followed by a touchdown protocol of 35 cycles of 95°C for 50 s, 65–55°C for 1 min (the annealing temperature decreased by 1°C each cycle for the first 10 cycles) and 72°C for 1 min. Samples were enzymatically cleaned with EXOSAP (Invitrogen, San Diego, CA), sequenced in both directions with Big Dye chemistry, and analyzed on an ABI 3730 automated sequencer at the Cornell University Life Sciences Core Laboratories Center for Genomics. All sequences have been deposited in GenBank (KC488896 - KC489085).

The resulting chromatograms were base called using the phred-phrap algorithm and assembled in CodonCode Aligner software (CodonCode Corp, Dedham, MA). All assembled sequences were trimmed and visually inspected. We included an additional 28 sequences from Willett et al. (1997) and 70 sequences from Maroja et al. (2009a) of mtDNA COI and constructed a phylogeny using MrBayes version 3.1.2 (Huelsenbeck and Ronquist 2001). To select the optimal substitution model, we used hierarchical likelihood ratio tests implemented in jModeltest version 0.1.1 (Posada 2008). To generate trees, we used the general time reversible model with invariant sites, gamma rates, and default priors (GTR + I + G), allowing the rate at each site to change over evolutionary history. We ran searches for ten million generations, sampling every 2,000 generations and discarded trees from the first 4,000,000 generations (burn-in time). We constructed a 50% majority-rule consensus tree from the remaining trees. The phylogenetic tree was rooted using three *G. rubens* sequences from Maroja et al. (2009a). MrBayes was run using the resources of the Cornell University Computational Biology Service Unit.

We used the Sequenom MassARRAY platform to genotype crickets for 12 mtDNA single nucleotide polymorphisms (SNPs). We collected 301 crickets across 46 sampling localities in Pennsylvania and 81 crickets from seven localities across the hybrid zone. A total of 66 crickets had previously been sequenced for the entire mtDNA COI gene (Table 1), and were used to validate our genotyping results. We assayed five SNPs in the mtDNA COI gene (site numbers 796, 952, 1036, 1204, 1382 from Willett et al. 1997) and seven mtDNA SNPs identified in Andrés et al. (2013). Multiplexed site-specific primers were used to amplify target DNA, followed by a single base extension of a primer immediately adjacent to the target SNP. The resulting product was analyzed using matrix-assisted laser desorption/ionization time-of-flight mass spectrometry (MALDI-TOF) and the mass difference of each possible SNP nucleotide allowed unambiguous genotyping. Assays were designed using the MassARRAY Assay Design version 4.0 (Sequenom, San Diego, CA, USA). Amplicon primer sequences, amplicon length, annealing temperature, extend primer sequence, and target SNP for each assay are listed in Table S2. Reactions were performed using iPLEX Gold chemistry at the Cornell Life Sciences Core Laboratories Center for Genomics and SNP genotypes were called using the Sequenom MassARRAY Typer Analysis version 4.0. All genotypes have been deposited in the Dryad Repository (http://dx.doi.org/10.5061/dryad.rr387). We constructed a phylogeny of the mtDNA SNPs using MrBayes as described above (generations: 10,000,000; sampling: 2,000; burn-in: 4,000,000; 50% majority-rule consensus).

### Morphological measurements

We characterized morphological traits that distinguish *G. firmus* and *G. pennsylvanicus* for crickets from nine allopatric populations (*G. firmus*: GUI, MAY, MOT, TOM, MET; *G. pennsylvanicus*: ITH, NBL, SCR, SCO) and from all collecting localities in our focal study area in Pennsylvania. We measured three morphological characters for each cricket: body length, femur length, and pronotal width. In addition, we measured the tegmina color in male crickets and the ovipositor length in female crickets (Fig. 2). Body length, femur length, and pronotum width all reflect overall size differences (*G. firmus* is typically larger than *G. pennsylvanicus*). Tegmina color (males) is lighter and ovipositor length (females) is greater in *G. firmus*. Ovipositor length is the character that most clearly differentiates the two species (Harrison and Arnold 1982; Harrison 1986). We also recorded the presence/absence of fully developed long hind-wings on both males and females; a polymorphic trait that affects flight ability. All size measurements were made to the nearest 0.1 mm using a pair of vernier calipers.

To measure the color of the male tegmina, we used a USB4000 spectrophotometer with a PX-2 pulsed xenon lamp (Ocean Optics, Dunedin, FL) to capture spectral reflectance. The probe was mounted in a metal stand at a 90^o^ angle 0.7 mm from the surface of the tegmina. For each male, we recorded and averaged spectral reflectance for three points near the center of the tegmina. We used the program SpectraSuite version 2.0 (Ocean Optics) to capture the wavelength readings. All spectral measurements were recorded as the percentage of reflected light relative to a Spectralon white standard (Ocean Optics). We restricted our analyses to wavelengths of 300–700 nm and used a segmental classification method to quantitate three aspects of color: brightness, chroma, and hue (Endler 1990) using the software program CLR (Montgomery 2008). We calculated total brightness (B) as R_300–700_, the summed reflectance from 300 nm to 700 nm. We also divided our reflectance data into four bins of 100 nm each, calculated the total brightness for each bin (B_r_ = 600–700, B_y_ = 500–600, B_g_ = 400–500, and B_b_ = 300–400) and then calculated chroma: √(B_r_−B_g_)^2^ + (B_y_−B_b_)^2^ and hue: arctan[(B_y_−B_b_)/B]/[(B_r_−B_g_)/B].

### Analysis of morphological data

We used principal component analysis (PCA) to explore variation in morphological data. We performed separate PCAs for male body size, tegmina color (brightness, chroma, and hue), and female body size using singular value decomposition of the scaled and centered morphological data with the function “prcomp” in R (R Core Development Team 2010). We performed a one-way analysis of variance (ANOVA) for each morphological trait (ovipositor length and the first principal components of male body size, male tegmina color, and female body size) to test for differences between allopatric *G. firmus* and *G. pennsylvanicus* populations. We used a linear discriminant analysis to determine how well each of these morphological traits classifies allopatric crickets. ANOVA and linear discriminant analyses were performed in R using the packages “stats” (R Core Development Team 2010) and “MASS” (Venables and Ripley 2002).

To identify crickets from within the Pennsylvania hybrid zone as *G. firmus*, *G. pennsylvanicus,* or admixed individuals, we used a fuzzy c-means clustering algorithm based on morphological traits that distinguish the two species in allopatric populations (male body size, tegmina color, female body size, and ovipositor length; see above analyses). We clustered individual crickets into these three classes using the “fanny” function from the R package “cluster” (Maechler et al. 2012). In contrast to hard clustering algorithms (such as *K*-means) in which data elements are divided into distinct clusters, fuzzy clustering allows data elements to belong to more than one cluster and assigns a corresponding set of membership levels (or membership coefficients). Because we were interested in identifying morphological clusters for the two parental species and admixed individuals, we assumed that there were two clusters (*k* = 2), which divided individuals into two morphological clusters (membership coefficients ≥ 0.1 or ≤ 0.9) and a third “fuzzy” or admixed cluster (membership coefficients < 0.1 and >0.9). The membership exponent (*r*) determines the degree of “fuzziness” in cluster assignment. A value of *r* = 1.0 assigns each data element to a single cluster and is equivalent to a classic *K*-means clustering, while values of *r* > 1.0 become increasingly fuzzy until all individuals are equally distributed among the *k* clusters (i.e., all belong to single “fuzzy” cluster). The proportion of individuals classified as admixed depends on the choice of *r*, but there is no clear rule for selecting *r* values. We used an approach, similar to other ecological studies (Schaefer and Wilson 2002; Gompert et al. 2010), of conducting separate fuzzy c-means clustering analyses for male morphology (male body size and tegmina color) and female morphology (female body size and ovipositor length) using *r* values ranging from 1.0 to 2.5. Values of *r* > 1.75 for male traits and >2.0 for female traits classified nearly all individuals as fuzzy. Therefore, we report cluster assignments using three values of *r* for male traits (1.25, 1.5, 1.75) and three values of *r* for female traits (1.5, 1.75, 2.0), all using *k* = 2 (Table 2). Values of *r* = 1.25 for males and *r* = 1.75 for females delineate cluster membership in a manner, which is consistent with classic *K*-means clustering algorithms and morphological indices that have been applied to these crickets (Harrison and Arnold 1982; Harrison 1986; Rand and Harrison 1989; Harrison and Bogdanowicz 1997) and were used for all further analyses.

**Table 2:** Morphological clustering. Proportion of crickets from the Pennsylvania hybrid zone assigned as *Gryllus pennsylvanicus (Gp)*, *G. firmus (Gf),* or admixed based on calculated squared Euclidean distances (fuzzy c-means clustering) using body size, tegmina color (males), and ovipositor length (females) assuming two clusters (*k* = 2). Higher membership coefficients (*r*) denote increased “fuzziness.”

Character	Cluster	*r* = 1.25	*r* = 1.50	*r* = 1.75	*r* = 2.0
Males	*Gp*	43.4%	37.0%	28.5%	–
(*N* = 424)	*Gf*	38.7%	33.7%	23.3%	–
	Admixed	17.9%	29.2%	48.1%	–
Females	*Gp*	–	37.9%	42.3%	32.8%
(*N* = 420)	*Gf*	–	48.6%	33.0%	25.6%
	Admixed	–	13.6%	24.7%	41.6%

### Environmental predictors of species distributions

We assessed 12 environmental variables for each of our 88 sampling sites at a 1-km scale for all variables. We calculated percent natural vegetation cover based on a 30-m resolution land-cover raster from circa 2005 (Homer et al. 2007). We considered urban, pasture, agriculture, silviculture, and recreational (e.g., golf-courses) land-cover types as non-natural. We calculated topographic complexity using the raster calculator feature in ArcGIS 9.3.1 (ESRI 2009), where each elevation pixel was assigned the variance of the neighbor pixels (Huaxing 2008). This metric provides significant information on habitat heterogeneity and microclimate turnover. We assessed physical soil characteristics for each sampling location (i.e., maximum% sand, silt, clay, and organic matter) using spatial data made available by Soil Data Mart (Soil Survey Staff 2012). We also recorded vegetation density (USGS and FAO 2000), latitude, elevation (Jarvis et al. 2009), human footprint (Sanderson et al. 2002), annual temperature (Bio 1; Hijmans et al. 2005), and annual precipitation (Bio 12; Hijmans et al. 2005).

We analyzed our data using simple linear regressions (standard least squares). We used this univariate approach to test the relationship of each explanatory variable with ovipositor length or morphological clustering membership coefficient. We then used model selection tests including all environmental variables and their interactions to find the combinations of variables that best explained ovipositor length and cluster membership. Competing models were ranked based on Akaike Information Criterion (AICc), and we reported the model with the highest goodness-of-fit for each run. Linear regression and automated model selection were conducted using JMP 10.0 (SAS 2012).

## Results

### Mitochondrial DNA

**Figure 3:**
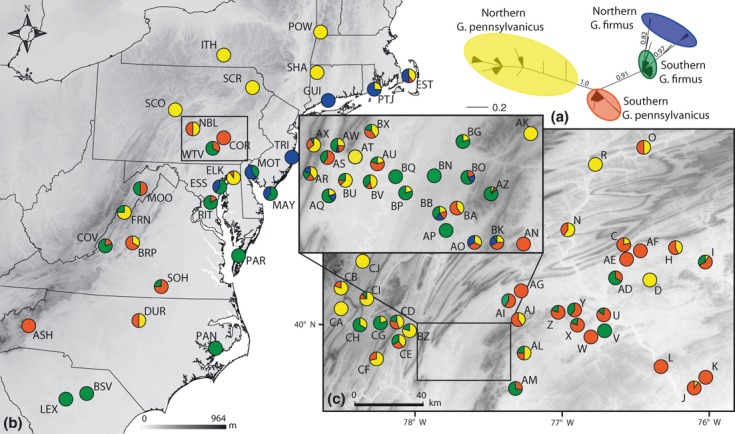
Mitochondrial DNA phylogeny and haplotype distribution for *Gryllus pennsylvanicus* and *G. firmus*. Yellow and orange represent northern and southern *G. pennsylvanicus* haplotypes and blue and green represent northern and southern *G. firmus* haplotypes, respectively. Each pie shows the proportion of crickets belonging to a mtDNA group and letters refer to the location (details in Table 1 and Table S1). a) Bayesian 50% majority-rule consensus tree of five mtDNA SNPs from cytochrome oxidase I (site numbers 796, 952, 1036, 1204, 1382 from Willett et al. 1997) and seven mtDNA SNPs identified in Andrés et al. (2013) (Table S2). Values on the branches correspond to the Bayesian posterior probabilities. The tree includes 81 crickets from 7 localities across the hybrid zone (Table S1) and 301 crickets from 46 sampling localities within the Pennsylvania hybrid zone. b) Distribution of mtDNA haplotypes across the hybrid zone. The rectangle highlights the location of the Pennsylvania study area. Shading indicates elevation in meters above sea level (m) with higher elevations represented by darker shades of gray. c) Detailed map of mtDNA haplotypes within Pennsylvania.

Phylogenetic analysis of the mtDNA COI gene produced a tree with four major groups, each group composed of conspecific crickets found primarily in circumscribed geographic areas: (1) northern *G. pennsylvanicus*, (2) southern *G. pennsylvanicus*, (3) northern *G. firmus*, and (4) southern *G. firmus* (Fig. S1). These four groups correspond to the haplotype groups identified by Willett et al. (1997) and Maroja et al. (2009a). Analysis of seven mtDNA SNPs identified the same four major haplotype groups, and two of these SNPs (Table S2, SNPs 448 and 554) were shared among the majority of *G. firmus* crickets (Fig. 3A). The proportion of crickets belonging to each mtDNA group for each sampling locality across the eastern United States and across our focal study area in Pennsylvania are presented in Fig. 3.

### Morphological data

**Figure 4:**
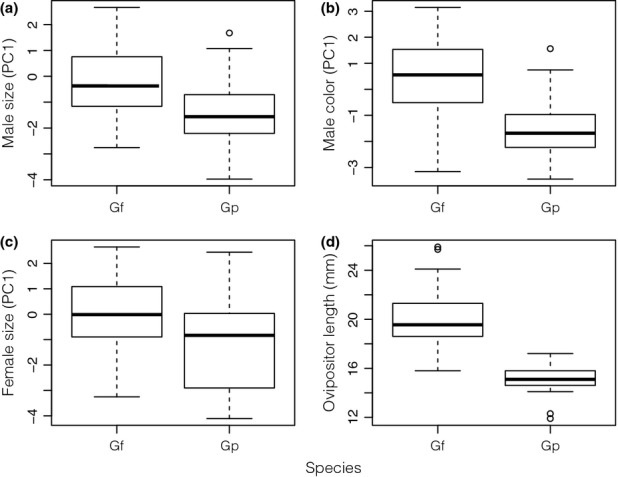
Morphological variation between allopatric populations of *Gryllus*. Boxplots of a) male body size, b) tegmina color, c) female body size, and d) ovipositor length for allopatric populations of *Gryllus firmus* (*N*_pops_ = 5, *N*_males_ = 72, *N*_females_ = 56) and *G. pennsylvanicus* (*N*_pops_ = 4, *N*_males_ = 63, *N*_females_ = 21).

The first principal component of male body size explained the majority of variation in body size among male crickets (87.7% ± 1.62) and all three measurements of male body size (body length, femur length, and pronotum width) had positive loadings on PC1. We used PC1 to represent male body size for all further analyses and we refer to this component as “male body size.” We found significant differences in male body size between *G. firmus* (*N* = 72, −0.19 ± 1.32) and *G. pennsylvanicus* (*N* = 63, −1.51 ± 1.22) (ANOVA: F_1,133_ = 35.94, *P* <0.0001), with *G. firmus* having larger body sizes (Fig. 4A). The first principal component of male tegmina color explained most of the variation in male tegmina color (75.0% ± 1.50) and all three measurements (tegmina brightness, chroma, and hue) had positive loadings on PC1. We refer to this component as “tegmina color.” Tegmina color differed significantly between *G. firmus* (*N* = 72, 0.42 ± 1.41) and *G. pennsylvanicus* (*N* = 63, −1.53 ± 1.04) (ANOVA: F_1,133_ = 81.48, *P* < 0.0001) with *G. firmus* having lighter tegmina (Fig. 4B). The variation in female body size was primarily explained by the first principal component (78.5% ± 1.53) and all three measurements (body length, femur length, and pronotum width) had positive loadings. This principal component is referred to as “female body size.” Female body size also differed significantly between *G. firmus* (*N* = 56, −0.01 ± 1.38) and *G. pennsylvanicus* (*N* = 21, −1.10 ± 1.85) (ANOVA: F_1,75_ = 7.81, *P* = 0.006) with *G. firmus* females having larger body sizes (Fig. 4C). Ovipositor length showed the largest difference between *G. firmus* (*N* = 56, 20.08 ± 2.11) and *G. pennsylvanicus* (*N* = 21, 15.15 ± 1.39) (ANOVA: F_1,75_ = 98.61, *P* < 0.0001) with *G. firmus* having longer ovipositors (Fig. 4D). The average size measurements for crickets from allopatric populations and collecting localities in the Pennsylvania hybrid zone are listed in Table S3 and Table S4 and morphological data have been deposited in Dryad Repository (http://dx.doi.org/10.5061/dryad.rr387).

Together, male body size and tegmina color correctly classified most individuals from allopatric populations as either *G. firmus* or *G. pennsylvanicus* (91.1% correctly classified). Eleven of 72 *G. firmus* crickets were misclassified as *G. pennsylvanicus* and one of 63 *G. pennsylvanicus* was misclassified as *G. firmus*. Pronotum width was the most important morphological character for classifying males, followed by tegmina hue (coefficients of linear discriminants: pronotum width = 0.979, tegmina hue = 0.795, body length = −0.387, femur length = −0.254, tegmina chroma = −0.122, tegmina brightness = 0.005). Female body size and ovipositor length together correctly classified all but a single *G. pennsylvanicus* (98.7% correctly classified). Ovipositor length was the most important morphological character for classifying females, followed by body length (coefficients of linear discriminants: ovipositor length = −0.600, pronotum width = 0.160, body length = 0.138, and femur length = −0.009).

**Figure 5:**
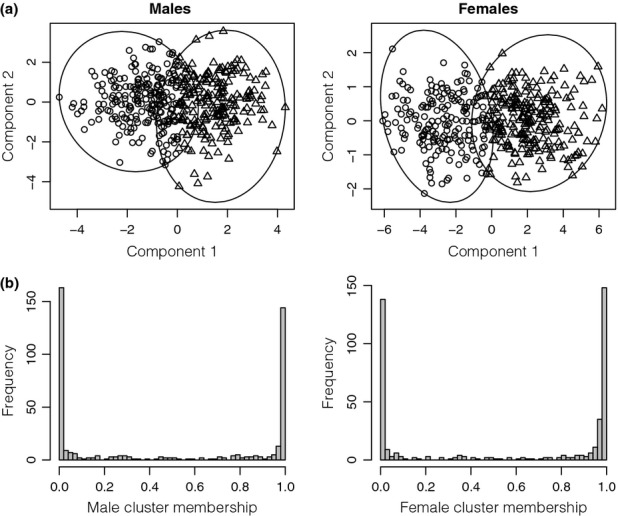
Morphological clusters for crickets from the Pennsylvania hybrid zone. a) Bivariate plot of morphological clusters (*k* = 2). Dissimilarities in morphological traits between individuals (males: body size and tegmina color; females: body size and ovipositor length) are calculated using squared Euclidean distances (fuzzy c-means) and represented by points in the plot using principal components. Ellipses indicate clusters of *Gryllus pennsylvanicus* (open circles) and *G. firmus* (open triangles). b) Distribution of fuzzy cluster membership coefficients for males and females (*G. pennsylvanicus* ≤0.10 and *G. firmus* ≥0.90).

**Figure 6:**
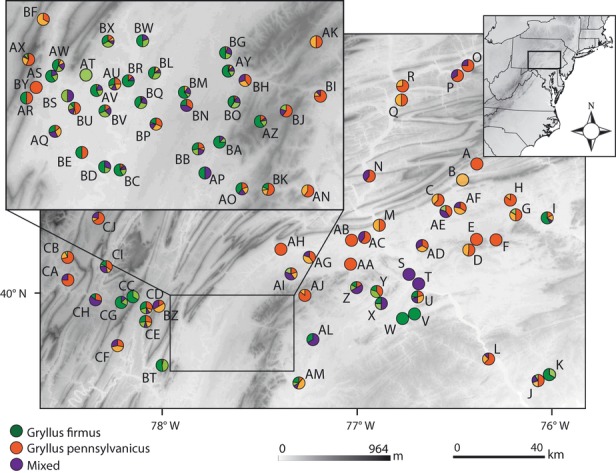
Distribution of *Gryllus pennsylvanicus* and *G. firmus* in the Pennsylvania hybrid zone. Proportion of individuals within each collecting locality identified as either *G. pennsylvanicus* (orange) or *G. firmus* (green) based on morphological clustering. Species assignments are based on morphological clusters; individuals with membership coefficients of 0 or 1.0 are dark orange and dark green and individuals with membership coefficients ≤0.10 and >0 or ≥0.90 and <1.0 are light orange and light green. Individuals with intermediate cluster coefficients <0.90 and >0.10 are purple.

Within the Pennsylvania hybrid zone, the majority of crickets were classified as either *G. firmus* or *G. pennsylvanicus* based on fuzzy c-means clustering (membership coefficients ≥0.90) (Fig. 5A). Values of *r* ranging from 1.25 to 1.75 and 1.50 to 2.0 for males and females, respectively, classified approximately 15–50% of crickets as intermediate between the two parental types (Table 2). The distribution of cluster membership coefficients was bimodal for both males and females, with slightly more males classified as intermediate and an overall greater number of crickets classified as *G. pennsylvanicus* (Fig. 5B) (see Fig. S2 for the distribution at each sampling locality). The proportion of individuals at each sampling locality classified as *G. pennsylvanicus* (membership coefficient ≤0.10), *G. firmus* (membership coefficient ≥0.90), or intermediate (membership coefficient >0.10 and <0.90) is depicted in Fig. 6. We found 2 collecting localities that were pure *G. pennsylvanicus* and 17 that were pure *G. firmus*. *Gryllus firmus* localities were more common in the Great Appalachian Valley between the Appalachians and the Blue Ridge, whereas *G. pennsylvanicus* were located mostly in the northeastern corner of our study area and in the large gap between the Reading Prong of the Northern Highlands and the Blue Ridge Mountains. There was evidence of admixture (morphologically intermediate individuals or shared mtDNA haplotypes) at 32 localities that could overall be characterized as predominantly *G. firmus* (14) or *G. pennsylvanicus* (18). There were 36 localities that contained both parental types and admixed individuals (Table 1).

**Figure 7:**
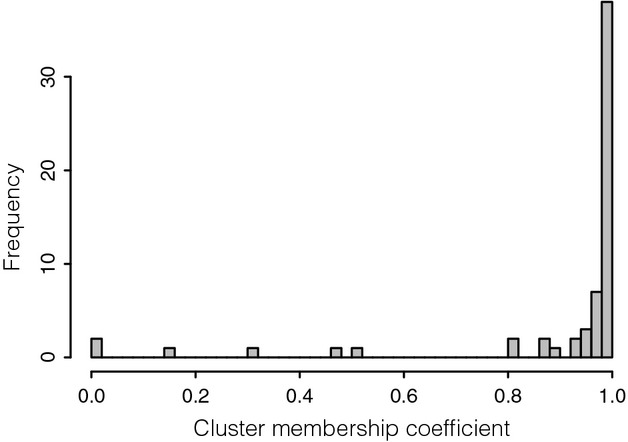
Distribution of cluster membership coefficients for long-wing morph crickets. There were a total of 61 long-wing morph crickets collected from 20 localities. The majority were *Gryllus firmus*, based on morphological clustering. Localities where long-wing crickets were found are in bold in Table 1.

Sixty-one of the 877 crickets we collected were long-wing crickets. These crickets were found at 20 localities of the 88 total localities sampled (Table 1). At most localities, we collected only one or two long-winged crickets (5–15%), but at three localities (CH, BV, BR), nearly a third of crickets were long-winged and at BG 77% of crickets were long-winged. The majority of the long-winged crickets were *G. firmus* (based on morphological clustering) (Fig. 7).

### Environmental predictors

#### Simple linear regressions

**Figure 8:**
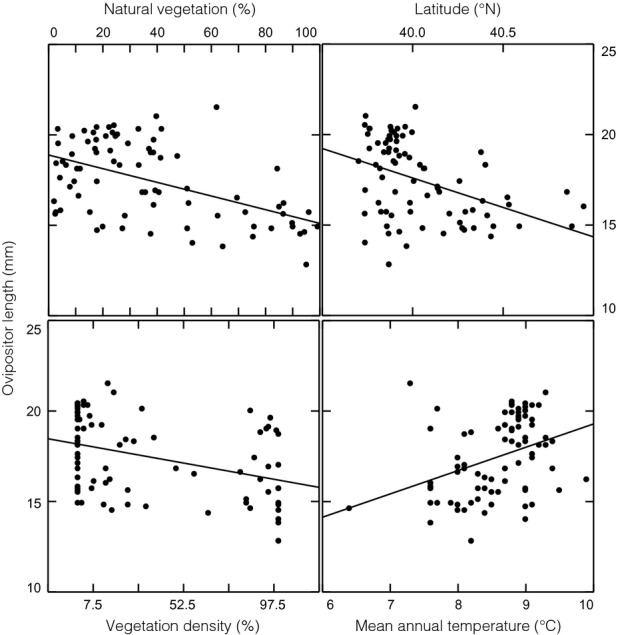
Simple linear regression of environmental variables. Scatterplots depicting the relationship between four environmental predictors and ovipositor length. Natural vegetation included all land types that were not urban, pasture, agriculture, silviculture, and recreational.

We found that natural vegetation, latitude, vegetation density, annual temperature, and annual rainfall best predicted ovipositor length in the Pennsylvania hybrid zone (Table 3, Fig. 8). Percent sand was only a marginally significant predictor of ovipositor length. Likewise, the same environmental variables were the best predictors of the morphological clustering membership coefficients: latitude, natural vegetation, annual temperature, annual rainfall, and vegetation density (Table 3). Percent sand and silt were only a marginally significant predictor of morphological cluster. The following environmental variables did not significantly influence either ovipositor length or cluster membership when considered in simple linear regressions: elevation, human footprint, topographic complexity, and soil physical characteristics for ovipositor (silt, clay, and organic content) and morphological cluster (clay, organic content).

**Table 3:** Results of simple linear regression for environmental variables influencing ovipositor length and morphological cluster membership coefficients in populations of *Gryllus firmus* and *G. pennsylvanicus* in Pennsylvania

Environmental predictor				
Ovipositor length	F_[1,77]_	*R*^2^	*P*	Beta
Natural vegetation	26.795	0.258	<0.0001	−0.037
Latitude	16.961	0.180	<0.0001	−3.254
Vegetation density	13.054	0.145	0.0005	−0.020
Annual temperature	11.570	0.130	0.0011	0.128
Annual rainfall	6.915	0.082	0.0103	−0.013
Sand	4.110	0.050	0.046	–
Elevation	2.139	0.027	0.147	–
Human footprint	0.027	0.003	0.600	–
Topographic complexity	0.048	0.001	0.826	–
Silt	2.367	0.029	0.128	–
Clay	0.022	0.0003	0.654	–
Organic content	1.766	0.022	0.187	–
Cluster membership	F_[1,86]_	*R*^2^	*P*	Beta
Natural vegetation	18.912	0.180	<0.0001	−0.005
Latitude	31.165	0.266	<0.0001	−0.625
Vegetation density	5.004	0.055	0.028	−0.002
Annual temperature	9.473	0.099	0.0028	0.018
Annual rainfall	5.998	0.065	0.016	−0.002
Sand	4.529	0.050	0.036	−0.005
Elevation	0.312	0.003	0.577	–
Human footprint	0.011	0.0001	0.916	–
Topographic complexity	0.366	0.004	0.547	–
Silt	4.335	0.048	0.040	−0.003
Clay	1.132	0.013	0.290	–
Organic content	1.736	0.019	0.191	–

##### Model selection: all possible models

When looking at all environmental factors simultaneously, natural vegetation and latitude best explained ovipositor length and morphological cluster membership coefficients (Table S5). The best model explaining ovipositor length included latitude, natural vegetation, and vegetation density as negative predictors, and also the interactions of natural vegetation with both latitude and vegetation density (Table 4). The best model explaining the cluster membership included latitude, natural vegetation, and organic matter as negative predictors, and also the interactions of natural vegetation with both latitude and organic matter (Table 4).

**Table 4:** General linear models testing simultaneously the effects of environmental factors on ovipositor length and morphological clustering membership coefficients for crickets from the Pennsylvania hybrid zone. Shorter ovipositors and lower morphological clustering membership coefficients represent *Gryllus pennsylvanicus* crickets. VIF = Variance inflation factor

Term	Beta	Std Beta	SE	*t*-Ratio	VIF	*P*
Ovipositor length						
Intercept	186.183	0.000	26.779	6.95	–	<.0001
Latitude	−4.181	−0.546	0.670	−6.24	1.277	<.0001
Vegetation density	−0.013	−0.249	0.005	−2.66	1.466	0.010
Natural vegetation	−0.016	−0.213	0.007	−2.16	1.622	0.034
Latitude × natural vegetation	0.050	0.215	0.021	2.45	1.287	0.017
Vegetation density × natural vegetation	−0.001	−0.272	0.000	−3.11	1.282	0.003
Morphological clustering membership coefficient						
Intercept	29.478	0.000	4.171	7.07	–	<.0001
Latitude	−0.717	−0.592	0.104	−6.89	1.276	<.0001
Organic matter	−0.042	−0.180	0.018	−2.32	1.046	0.022
Natural vegetation	−0.004	−0.363	0.001	−4.59	1.079	<.0001
Latitude × natural vegetation	0.012	0.331	0.003	3.900	1.244	0.0002
Organic matter × natural vegetation	0.001	0.238	0.001	3.000	1.089	0.003

Whole model statistics: ovipositor length (F_[5,73]_ = 18.861; *R*^2^ = 0.563; *P* < 0.0001); morphological clustering membership coefficients (F_[5,82]_ = 18.084; *R*^2^ = 0.524; *P* < 0.0001).

### Discussion

The field cricket hybrid zone in Pennsylvania is a mosaic of genetically and morphologically distinct populations. The distribution of both genetic and morphological types is highly heterogeneous and cannot be explained as a function of distance across the hybrid zone; there is no clear clinal pattern of variation for any trait, but rather a patchwork of populations. This is similar to the patterns seen in other regions of the hybrid zone in Virginia (Harrison and Arnold 1982) and Connecticut (Harrison 1986; Harrison and Rand 1989; Rand and Harrison 1989) (Table 5). Mosaic hybrid zones occur when the ecological settings and/or geography in the area of overlap are heterogeneous or complex, and species distributions are determined by environmental selection (e.g., Harrison 1986; Howard 1986; Harrison and Rand 1989; Bridle et al. 2001; Ross and Harrison 2002; Bierne et al. 2003; Vines et al. 2003; Ross et al. 2008). In Pennsylvania, we find an association between species distribution and natural habitat; *G. pennsylvanicus* occupies natural habitat along forest edges and natural clearings, whereas *G. firmus* occupies more disturbed areas in agricultural and suburban environments. Hybridization and introgression occur across patch boundaries; there is evidence of substantial admixture both in morphological characters and mtDNA, over a broad geographic area.

**Table 5:** Summary of the ecological barriers to gene exchange for three well-characterized regions of the hybrid zone between the field crickets, *Gryllus pennsylvanicus* and *G. firmus*. Included is the spatial scale each region was sampled (Scale), the structure of the hybrid zone at that spatial scale (Structure), and the ecological barriers identified as contributing to the hybrid zone structure (Ecological barrier)

Region	Scale	Structure	Ecological barrier		Citations
Connecticut	Regional	Clinal	Ecogeographic isolation	*G. pennsylvanicus* occupies inland and upland sites (northwest); *G. firmus* occupies costal and lowland sites (central coast)	Harrison and Arnold 1982; Harrison 1986; Harrison and Rand 1989;
	Local	Mosaic	Habitat isolation	*G. pennsylvanicus* associated with loamy soils; *G. firmus* associated with sandy soils	Harrison 1986; Rand and Harrison 1989; Harrison and Bogdanowicz 1997;
	Fine	Clinal	–	–	Ross and Harrison 2002;
Virginia	Regional	Clinal	Ecogeographic isolation	*G. pennsylvanicus* occupies upland sites (west); *G. firmus* occupies lowland sites (east)	Harrison and Arnold 1982;
	Local	Mosaic	Temporal isolation	*G. firmus* has a slower development time and adults emerge later in the fall	Harrison and Arnold 1982; Harrison 1985;
Pennsylvania	Regional	Clinal	Ecogeographic	*G. pennsylvanicus* occupies upland sites (north and west); *G. firmus* occupies lowland sites (south and east)	Harrison and Arnold 1982
	Local	Mosaic	Habitat isolation	*G. pennsylvanicus* associated with natural habitat; *G. firmus* associated with disturbed habitat	This study

#### Broad scale distribution of G. firmus and G. pennsylvanicus: morphology and mtDNA

Our sampling of *G. firmus* and *G. pennsylvanicus* revealed the same four major mtDNA haplotype groups that were found by Willett et al. (1997) and Maroja et al. (2009a). *Gryllus pennsylvanicus* consists of two major clades (northern and southern) and *G. firmus* has a distinct southern clade, and a northern group that is distinguishable from the other three well-supported clades (Fig. S1). Given that we find all four mtDNA haplotypes at three collecting localities in central Pennsylvania (BB, AR, BK), this region appears to represent the geographic divide between northern and southern groups of each species (Fig. 3). The hybrid zone appears to extend further north than previously described (Harrison and Arnold 1982; Harrison 1986; Maroja et al. 2009a). We found crickets in Rhode Island and Massachusetts that have introgressed mtDNA and the Massachusetts locality contains both parental types. To the north, we found only pure *G. pennsylvanicus* populations. Thus, Massachusetts may represent the northern range limit of *G. firmus*.

The relationship among the mtDNA groups has not been resolved with sequence data from the mtDNA COI gene. Willett et al. (1997) found five equally parsimonious tree topologies for these groups and in all cases, either northern or southern *G. pennsylvanicus* clades were the basal group, with the southern *G. pennsylvanicus* clade having the greatest haplotype diversity. Additional genotyping of seven mtDNA SNPs identified two nucleotide positions in the ATPase6 and COIII genes that are shared between northern and southern *G. firmus* (Fig. 3A). This suggests that an ancestral cricket lineage split into two daughter lineages, one that became either northern or southern *G. pennsylvanicus* and a second that split into the other *G. pennsylvanicus* clade and *G. firmus*. *Gryllus firmus* has subsequently diverged into northern and southern groups.

#### Patches of natural habitat contribute to the mosaicism of the Pennsylvania hybrid zone

Throughout their ranges, both *G. pennsylvanicus* and *G. firmus* can be found in disturbed habitats along roadsides, in fields and pastures and around human settlement. Yet, in the Pennsylvania hybrid zone, we see an association between species distribution and natural habitat; *Gryllus pennsylvanicus* occupies natural habitat along forest edges and clearings, while *G. firmus* occupies disturbed habitat near human settlement and agriculture (Table 4, Fig. 8). In the Pennsylvania study area, there is more natural habitat in the Appalachian Mountains to the north, which can explain why we also see a correlation between the distribution of *G. pennsylvanicus* and higher latitudes, greater vegetation density, lower temperatures, and more rainfall. However, we also find *G. pennsylvanicus* crickets in patches of natural habitat further south along the Blue Ridge Mountains (e.g.*,* AJ, AK, AN, BI) and near rivers, lakes, and parks in the large gap between the Blue Ridge Mountains and the Reading Prong of the Northern Highlands (e.g., C, D, H, L, Z, Y). Likewise, *G. firmus* occurs further west into the Appalachian Mountains than earlier surveys of the hybrid zone suggested (Harrison and Arnold 1982; Maroja et al. 2009a).

We found a high proportion of crickets with both *G. firmus* morphology and mtDNA haplotypes in the Great Appalachian Valley and the small valleys within the Appalachians. *Gryllus firmus* likely expanded north through these corridors and crossed the steep mountain ridges along roadways through natural water and wind gaps (CG, CH, CD, CE, BT). In some areas, it appears that human disturbance has facilitated the persistence of *G. firmus* in otherwise heavily forested, natural habitats (CC and AZ).

Given that both cricket species seem well adapted to disturbed areas, it is unlikely that either performance in or preference for disturbed habitat restricts the distribution of *G. pennsylvanicus*. It is more likely that *G. firmus* is either less well suited for the habitat characteristic of *G. pennsylvanicus*' range or that *G. firmus* is particularly well suited for disturbed habitat and is a better colonizer. Typically, field crickets have short hind-wings and are incapable of flight. Daily movements such as feeding, reproduction, and predator avoidance are accomplished by walking. But in both species, individuals can be found with long hind-wings and fully developed flight muscles, capable of long-distance dispersal (Alexander 1968). The development of long-winged morphs is triggered by environmental variables such as temperature, population density, and resource availability (Harrison 1980). However, *G. firmus* and *G. pennsylvanicus* have both intra- and interspecific variation in their proportions of long-winged individuals that can be explained in part by genetic differences (Harrison 1979). *Gryllus pennsylvanicus* has on average of 4% long-winged individuals (Alexander 1968; Harrison 1979), whereas the frequency for *G. firmus* has been reported to be as high as 10–30% in some populations (Veazey et al. 1976; Harrison 1979). Wing dimorphism is thought to be particularly prevalent in *G. firmus* because its natural habitat is often highly disturbed and ephemeral (e.g., sand dunes, beach grass, and under shoreline debris) and may necessitate frequent dispersal among habitat patches (Roff 1990). Indeed, approximately 82% of the long-wing crickets identified in this study were *G. firmus,* whereas only 3% were *G. pennsylvanicus* (the remainder had fuzzy morphological membership coefficients) (Fig. 7). Wing dimorphism averaged 5–10% in *G. firmus* populations, but ranged as high as 30–75% at some collecting localities. In all cases, long-winged crickets were found at localities with high population densities and in disturbed habitats (Table 1). This suggests that *G. firmus* is both capable of thriving in disturbed habitats and may have a greater propensity for long-distance dispersal.

The association of *G. pennsylvanicus* with natural habitat and *G. firmus* with disturbed habitat is likely to be a major factor contributing to the mosaic structure of the hybrid zone in Pennsylvania. However, the environmental variables associated with hybrid zone structure are very different between Pennsylvania and other regions of the hybrid zone (Table 5). In Connecticut, soils vary over very short distances from loam to sand, and there is a clear association between species distributions and soil type; *G. firmus* occur on sandy soils and *G. pennsylvanicus* on loam (Harrison 1986; Rand and Harrison 1989; Ross and Harrison 2002). This may reflect adaptive differences in ovipositor length (*G. firmus* has a relatively longer ovipositor) for egg placement in different soil types (Masaki 1979; Bradford et al. 1993). In contrast, Pennsylvania soils are predominantly clay (≥20% clay) and we saw no correlation between soil properties and species distributions (Table 3). In Virginia, there is also no association between species distribution and soil type. Instead, elevation and temperature appear to contribute to hybrid zone structure; *G. pennsylvanicus* occupies high elevation sites in the Appalachian Mountains, while *G. firmus* is primarily in the lowlands. This is likely driven by differences in development time. *Gryllus firmus* from Virginia develop more slowly than *G. pennsylvanicus* (both in the field and in the lab) resulting in offset adult emergence (Harrison 1985). There are likely climatic life cycle shifts in *G. firmus*; southern crickets develop quickly and have multiple generations per year, but in mid-latitudes (Virginia), development may slow to accommodate only one generation per year and at even higher latitudes (Connecticut), shorter growing seasons may again favor faster development rate (Fulton 1952; Alexander 1968; Walker 1980; Harrison 1985). In both Connecticut and Pennsylvania, crickets appear to emerge synchronously, and there is no evidence that temporal isolation contributes to hybrid zone structure.

#### Patterns of admixture suggest strong pre-zygotic barriers

In mosaic hybrid zones, the patchy distribution of parental types results in extensive contact throughout the zone. Hybridization and introgression occur across patch boundaries or in intermediate habitats. In the Pennsylvania hybrid zone, there is a patchy distribution of natural and disturbed habitat and a corresponding distribution of *G. pennsylvanicus* and *G. firmus*. There are numerous opportunities for contact in areas where there are transitions in patch type: along mountains slopes, intersecting roadways, and near encroaching human development. Despite these opportunities for hybridization, we found that the majority of collecting sites are predominantly composed of a single parental type and a few individuals with intermediate morphologies that may be admixed (most likely backcrosses) (Fig. 6). Indeed, many of the crickets from sites with intermediate *G. firmus* morphologies had *G. pennsylvanicus* mtDNA haplotypes, suggesting that morphology is a good indicator of admixture. Each of these individual populations has an L-shaped distribution of morphological cluster membership (Fig. S2), but the combination of these predominantly *G. firmus* and *G. pennsylvanicus* populations results in an overall bimodal distribution within the hybrid zone (Fig. 5B). A few collecting localities contained both parental types, and a number of sites appeared to be mixed (containing both parental types and morphologically intermediate individuals).

The topographic complexity of the region may also explain why the hybrid zone appears broader across the central Appalachian Mountains than early surveys of the hybrid zone suggested (Harrison and Arnold 1982; Willett et al. 1997; Maroja et al. 2009a). The sharp transitions between forested mountains and populated valleys increase the patchiness of natural habitat and could increase the extent of hybridization. In addition, increased human disturbance as a result of suburban expansion, agriculture, and resource extraction is likely expanding the area of contact by increasing suitable habitat for *G. firmus*. Contact in some of these areas may even be very recent. For instance, the occurrence of *G. firmus* along the Pennsylvania turnpike (CG, CH, CD) in relatively discrete locations suggests that *G. firmus* may have only begun occupying these high elevation sites in recent decades.

Although we found evidence of substantial admixture both in morphological characters and mtDNA over a broad geographic area, the two species remain distinct. Most admixed individuals are morphologically like one or the other parental type (Fig. 5B), and there are few intermediate individuals. Given that F_1_ hybrids are viable and fertile in the lab, this suggests that strong pre-zygotic barriers are operating in this portion of the hybrid zone, a pattern consistent with characterizations of other regions of the hybrid zone in Virginia (Harrison and Arnold 1982) and Connecticut (Harrison 1986; Harrison and Bogdanowicz 1997). Barriers involved in behavioral isolation (Harrison 1986; Harrison and Rand 1989; Maroja et al. 2009b) and post-mating barriers that prevent fertilization (Harrison 1983; Larson et al. 2012) appear to be consistent across these different regions of the hybrid zone. In contrast, the ecological barriers that likely contribute to the hybrid zone's mosaic structure appear to vary between geographic regions (Table 5). In Connecticut, crickets are associated with different soil types, whereas in Virginia, crickets occur at different elevations and are temporally isolated due to differences in development time. In Pennsylvania, we found that the extent of natural habitat best explains the distribution of the two cricket species. This variation can have important consequences for patterns of introgression among different regions of the hybrid zone; genes involved in ecological isolation may vary in the extent of introgression in different environmental contexts (Harrison 1990; Payseur 2010). Interpreting patterns of variable introgression requires a clear understanding of the environmental context of species interactions (Nolte et al. 2009; Teeter et al. 2010; Macholan et al. 2011; Janousek et al. 2012). Species boundaries have been described as semipermeable, and this permeability varies not only across different genomic regions but also among different geographic areas and ecological contexts (Rand and Harrison 1989). Characterizing multiple regions within a hybrid zone is therefore critical for understanding hybrid zone dynamics, and gaining insights into the nature of species boundaries.

## Supporting Information

**Figure S1.** Bayesian 50% majority-rule consensus tree of the mtDNA gene cytochrome oxidase I sequence data. Yellow and orange represent northern and southern *G. pennsylvanicus* haplotypes and blue and green represent northern and southern *G. firmus* haplotypes, respectively. Values on the branches correspond to the Bayesian posterior probabilities. The tree includes all samples from Willett et al. (1997) and Maroja et al. (2009a), plus an additional 130 crickets from 13 localities across the hybrid zone (Table S1) and 119 crickets from 31 localities within the Pennsylvania hybrid zone (Table 1).

**Figure S2.** Distribution of morphological clustering membership coefficients for males (*r =* 1.25) and females (*r* = 1.5) for each collecting locality. Dissimilarities in morphological traits between individuals (males: body size and tegmina color; females: body size and ovipositor length) are calculated using squared Euclidean distances (fuzzy c-means). Collecting localities are ranked by the average clustering index, with *Gryllus firmus* localities at the top-left and *G. pennsylvanicus* localities at the bottom-right.
